# DNA Methylation of *iNOS*, *eNOS, TP53* and LINE‐1 in Gingival Tissues of Cigarette Smokers, Heat‐Not‐Burn Smokers and Never Smokers: An Exploratory Study

**DOI:** 10.1002/cre2.70141

**Published:** 2025-05-19

**Authors:** Giulio Rasperini, Michele Codari, Alessandra Moiana, Letizia Tarantini, Valentina Bollati, Gionata Bellucci, Dario Consonni, Lena Larsson, Farah Asa'ad

**Affiliations:** ^1^ Department of Biomedical Surgical and Dental Sciences University of Milan Milan Italy; ^2^ Fondazione IRCCS Ca' Granda Ospedale Maggiore Policlinico Milan Italy; ^3^ EPIGET‐Epidemiology, Epigenetics and Toxicology Lab, Department of Clinical Sciences and Community Health University of Milan Milan Italy; ^4^ Epidemiology Unit, Fondazione IRCCS Ca' Granda Ospedale Maggiore Policlinico Milan Italy; ^5^ Department of Oral Biochemistry, Institute of Odontology, The Sahlgrenska Academy University of Gothenburg Göteborg Sweden

**Keywords:** DNA methylation, eNOS enzyme, iNOS enzyme, LINE‐1 elements, oral mucosa, smoking, *TP53* gene

## Abstract

**Objectives:**

In this exploratory study, the influence of traditional cigarette smoking and heat‐not‐burn device smoking (HnB) on DNA methylation of cancer‐associated genes in smokers compared to never smokers was evaluated.

**Materials & Methods:**

Twenty‐eight healthy individuals undergoing dental care were enrolled into three groups: (i) 10 traditional smokers, (ii) 8 HnB smokers, and (iii) 10 never smokers. Gingival biopsies were obtained, and DNA methylation analysis was performed by pyrosequencing for the cancer‐associated genes: *iNOS*, *eNOS* and *TP53*. LINE‐1 sequence was selected for genome‐wide methylation readout.

**Results:**

HnB smokers exhibited approximately a 13% higher *iNOS* methylation compared to both traditional smokers and never smokers (*p* = 0.004 in a crude analysis and *p* = 0.01 in a multivariable linear regression model adjusted for gender and age). However, HnB smoking did not influence the DNA methylation levels of *TP53, eNOS*, and LINE‐1 in gingival tissues, as these were comparable to the levels observed in traditional smokers and never smokers.

**Conclusions:**

HnB device smoking increased DNA methylation levels of *iNOS* gene, which might indicate a decreased iNOS expression in HnB smokers, compared to traditional smokers and never smokers. Implications of the observed *iNOS* methylation status in the development of oral cancer needs to be investigated in future studies.

## 1 Introduction

DNA methylation is a major epigenetic mechanism that plays a critical role in regulating gene expression by adding methyl groups to cytosine bases situated next to guanine bases in CpG islands or sites. This modification is associated with gene silencing, while transcriptionally active genes are associated with low levels of methylation (Robertson and Wolffe 2000; Bird 2002).

In the context of head and neck squamous cell carcinoma (HNSCC), changes in DNA methylation have been associated with smoking history (Smith et al. 2007). Interestingly, it was demonstrated that smoking results in alterations in long interspersed element‐1 (LINE‐1) methylation in the oral epithelium, resulting in hypo‐ and hypermethylated loci (Wangsri et al. 2012). It must be noted that hypomethylated LINE‐1 may possess carcinogenesis potential (Wangsri et al. 2012).

Nitric oxide (NO) is another critical player in cancer biology, acting as a “double‐edged sword.” While NO is a key signaling molecule under physiological conditions, its dysregulated synthesis has been implicated in cancer progression (Crowell et al. 2003; Choudhari et al. 2013). NO is synthesized by three nitric oxide synthase (NOS) enzymes: neuronal (nNOS/NOS1), inducible (iNOS/NOS2), and endothelial (eNOS/NOS3), each encoded by a distinct gene (Khan et al. 2020). Among these, iNOS generates the highest levels of NO in a calcium‐independent manner (Goligorsky et al. 2004; Thomas et al. 2008). Increased iNOS levels have been linked to oral squamous cell carcinoma (OSCC) (Connelly et al. 2005), which is the most common type of HNSCC (Jagadeesan et al. 2024), while *eNOS* gene polymorphisms were shown to influence the levels of OSCC risk in the Serbian population (Carkic et al. 2020).

The tumor suppressor protein p53, encoded by the *TP53* gene (Yang et al. 2015), is another key molecule in OSCC. p35 acts as the “guardian of the genome”, by preventing genome mutation, and thus ensures its stability (Strachan and Read 1999). In its wild‐type form, p53 is a major tumor suppressor, which is protective against various types of cancers (Mercer 1992), while the mutant p53 becomes a tumor‐promoting factor (Liu et al. 2010). Recent findings have revealed a novel tumor‐suppressive function of wild‐type p53, through the repression of LINE‐1 transposons, whose activation can drive oncogenesis (Tiwari et al. 2020). On the other hand, LINE‐1 expression in cancer correlates with p53 mutation (McKerrow et al. 2022). Interestingly, high frequency in *TP53* gene mutation has been reported in OSCC (Li and Zhang 2015). The interplay between iNOS and p53 in OSCC highlights a complex relationship, where increased iNOS expression alters p53 function, promoting tumorigenesis (Yang et al. 2015).

Based on all these findings and given the emerging evidence of epigenetic alterations in cancers, this study investigates the DNA methylation levels of *iNOS*, *eNOS, TP53* and LINE‐1in the oral mucosa of never smokers, traditional cigarette smokers, and Heat‐not‐Burn (HnB) device users. In recent years, HnB smoking devices, marketed as a less harmful alternative to traditional smoking, have gained popularity (Simonavicius et al. 2019). These devices heat tobacco without combustion, potentially reducing the exposure to toxicants (Simonavicius et al. 2019). However, little is known about their effects on DNA methylation in cancer‐related genes.

By comparing these groups, we aim to determine whether different smoking habits differentially influence the methylation of cancer‐related genes, potentially altering their expression and contributing to carcinogenesis.

## 2 Materials and Methods

This study was approved by the ethical committee of the University of Milan, Italy, (Comitato Etico Milano Area 2, Fondazione IRCCS Ca' Granda Ospedale Maggiore Policlinico, Palazzo Uffici—20122 Milan), and was conducted during the period between March 2021 and July 2021.

### 2.1 Study Participants and Inclusion Criteria

From the pool of patients attending the Dental Clinic of the Foundation IRCCS Ca' Granda Policlinico—University of Milan, Italy, 28 individuals without periodontitis or other advanced dental diseases, such as advanced dental caries, were enrolled in three groups: 10 never smokers, 10 current traditional cigarette smokers (smoking 10 or more cigarettes/day), and 8 HnB device smokers (smoking 10 or more cigarettes/day). A threshold of 10 cigarettes per day is commonly used to distinguish between light smokers and moderate/heavy smokers.

Verbal and written informed consent were obtained from all participants after explaining the objectives of the study.

Participants who fit the following inclusion criteria were enrolled in the study:

1. Individuals ≥ 18 years of age.
2. Individuals of Caucasian origin.
3. Individuals who are systemically healthy, i.e., without any reported systemic diseases.
4. For never smokers: never smoked.
5. For traditional and HnB smokers: smoking for at least 2 years when enrolled in the study.

The exclusion criteria were as follows:

1. Pregnancy.
2. Individuals who reported intake of nonsteroidal anti‐inflammatory drugs (NSAIDs) or antibiotics within 6 months preceding biopsy harvesting.

### 2.2 Biopsy Harvesting Procedure

Gingival biopsies were harvested from patients who needed a crown lengthening procedure or wisdom tooth extraction. All the samples were collected by the same operator (A.M.). The samples were harvested after local anesthesia by means of a tissue punch for biopsy of 3 mm diameter, at the papilla area between the molar and premolar on the palatal aspect.

### 2.3 DNA/RNA Extraction & DNA Methylation Assay

Freshly harvested tissues were collected in Allprotect Tissue Reagent (Qiagen, USA) to stabilize DNA and stored at 2°C–8°C as recommended in the manufacturer's instructions until analysis. Each vial was assigned a code to keep the patient anonymous. DNA/RNA extraction was performed using the AllPrep DNA/RNA Mini Kit (Qiagen, USA) following the manufacturer's instructions.

The DNA methylation analysis was performed in the EPIGET Lab (University of Milan, Italy) using bisulfite‐PCR‐pyrosequencing as previously described (Asa'ad et al. 2017). Primers used for DNA methylation analysis and PCR cycling conditions are shown in (Table 1).

**Table 1:** Primers for DNA methylation analysis and PCR cycling conditions.

Sequence ID	Forward Primer(5′ to 3′)	Reverse Primer(5′ to 3′)	Sequencing Primer(5′ to 3′)	Sequence analyzed*(5′ to 3′)	Annealing conditions	Fragment size (bp)
***Repetitive element methylation analysis***						
**LINE‐1**	TTTTGAGTTAGGTGTGGGATATA	Biotin‐AAAATCAAAAAATTCCCTTTC	AGTTAGGTGTGGGATATAGT	TTC/TGTGGTGC/TGTC/TG	50°C for 30”	146
***Gene‐specific methylation analysis***						
***iNOS***	AATGAGAGTTGTTGTTGGGAAGTGTTT	Biotin‐CCACCAAACCCAACCAAACT	TAAAGGTATTTTTGTTTTAA	C/TGATTTTC/TGGGTTTTTTTTTATTTTG	Touch down 60°–54°C, then 50°C 45”	212
***eNOS***	TGTAGTTTTAGGGTTTTGTTGGA	Biotin‐CCCCTATCCCATACACAAT	TATTAGTTTTAGTTTTTATA	GC/TGGAATTTAGGC/TGTTC/TGGTTTTTT	72°C for 60”	169
***TP53***	BIO‐TTAGGAGTTTATTTAATTTAGGGAAG	TATCCAACTTTATACCAAAAACCTC	TCCAAAAAACAAATAACTACTAAACTC	CG/AAAAACACTTTACG/ATTCG/AAACTAAAAACG/ATACTTT	57°C 1′	220

* Nucleotides at which DNA methylation was measured are underlined.

The DNA methylation level of 3 candidate genes *P53*, *iNOS*, and *eNOS,* and LINE‐1 repetitive element sequence was quantified. The methylation level at CpG positions was determined as the number of methylated cytosines divided by the sum of methylated and unmethylated cytosines, multiplied by 100% (% 5‐methylcytosine). Every sample was tested twice for each marker to confirm reproducibility and to increase the precision of the results. The selected CpG positions are shown in (Table 2).

**Table 2:** The analyzed CpG positions.

Gene	Chromosome	Promoter		Amplicon		CpGs
		Start	End	Start	End	
***eNOS***	7	150321305	150321905	150321535	150321703	150321595 (pos1); 150321606 (pos2); 150321610 (pos3)
***iNOS***	17	23149861	23150461	23149873	23149990	23149929 (pos1) 23149936 (pos2)
***TP53***	17	7531143	7531743	7531409	7531628	7531486 (pos1); 7531473 (pos2); 7531469 (pos3); 7531458 (pos4)

### 2.4 Statistical Analysis

Mean methylation levels were analyzed with Kruskal–Wallis tests. Multivariable linear regression models were then fitted to compare methylation levels across the three groups, adjusting for gender and age (continuous). Statistical analyses were performed by using Stata 17 (StataCorp. 2021).

## 3 Results

Twenty‐eight participants (16 males, 12 females) were included in this study, distributed into 10 never smokers (five males, five females), 10 traditional cigarette smokers (six males, four females), and 8 HnB device smokers (five males, three females) (Table 3). The age range of all the study participants was 21–80 years. HnB device smokers were on average younger than the other groups.

**Table 3:** Characteristics of the study participants.

Demographics	Never smokers (*n* = 10)	HnB smokers (*n* = 8)	Traditional cigarette smokers (*n* = 10)	*p*‐value
**Males/Females**	5/5	5/3	6/4	0.85
**Age (years) (Mean **± *SD* **)**	47.8 ± 20.6	39.1 ± 10.8	49.9 ± 18.6	0.54

**Figure 1:**
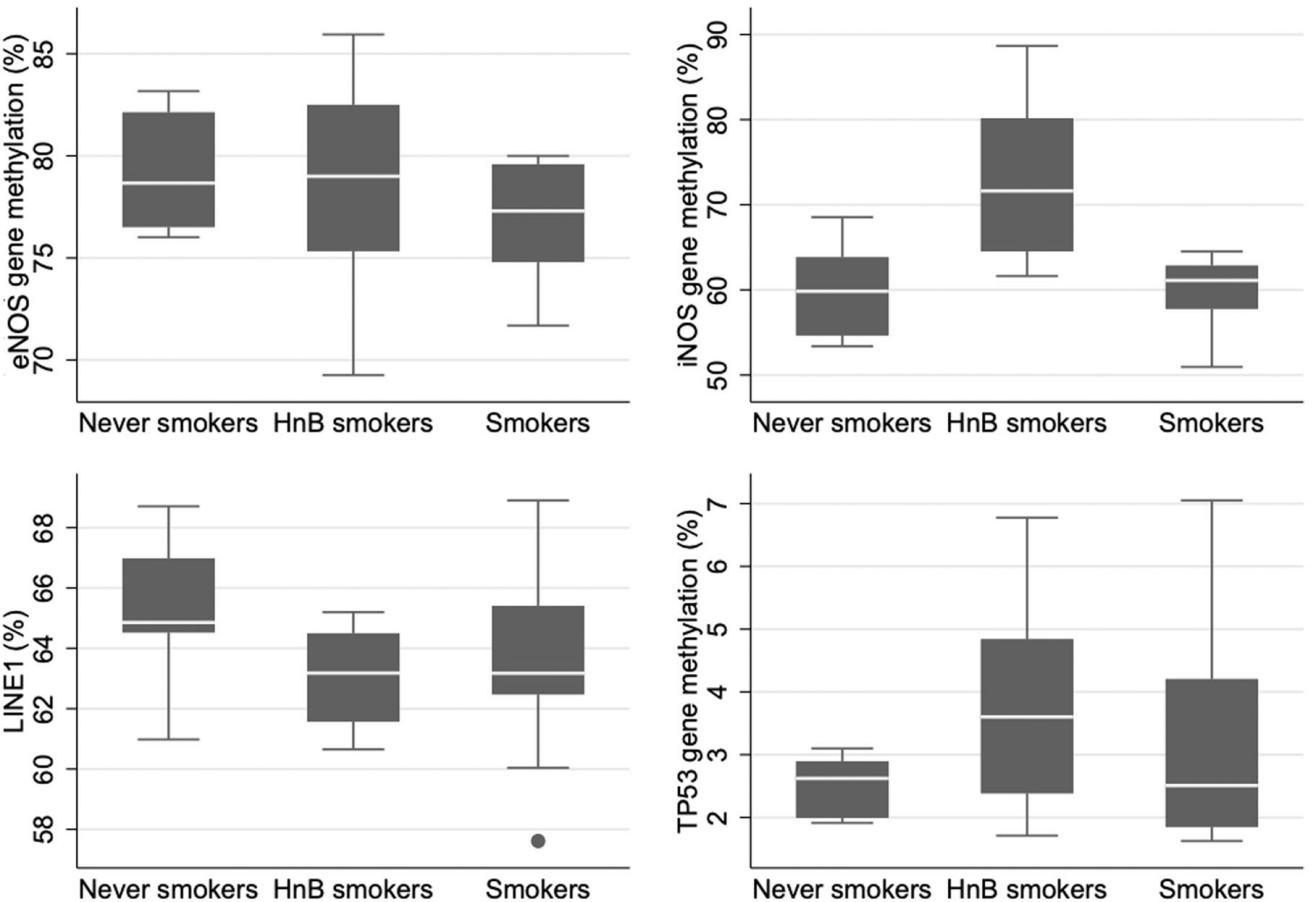
Box and whiskers plots showing methylation (%) of *eNOS*, *iNOS*, *TP53* genes and LINE‐1 in 28 patients (10 never‐smokers, 8 Heat‐not‐Burn (HnB) smokers, and 10 smokers of traditional smokers. The box contains 50% of the patients; the white line within the box represents the median value; box and whiskers contain about 85% of patients.

Regarding the DNA methylation levels, our results show that HnB smokers exhibited approximately a 13% higher *iNOS* methylation (72.9 ± 9.8) compared to both traditional smokers (59.8 ± 4.7) and never smokers (59.6 ± 5.2) (*p* = 0.004 in a crude analysis and *p* = 0.01 in a multivariable linear regression model adjusted for gender and age) (Table 4, Figure 1). However, HnB smoking did not influence the DNA methylation levels of *TP53, eNOS*, and LINE‐1 in gingival tissues, as these were comparable to the levels observed in traditional smokers and never smokers.

**Table 4:** Methylation of *eNOS*, *iNOS*, *TP53* genes and LINE‐1 across groups.

Methylation percentage (Mean ± SD)					
Gene/Sequence	Never smokers	HnB smokers	Traditional cigarette smokers	*p* value*	*p* value **
***eNOS***	79.1 ± 2.7	78.8 ± 5.4	76.9 ± 2.9	0.27	0.40
***iNOS***	59.6 ± 5.2	72.9 ± 9.8	59.8 ± 4.7	0.004	0.001
***TP53***	2.5 ± 0.5	3.8 ± 1.7	3.2 ± 1.8	0.50	0.28
**LINE‐1**	65 ± 2.4	63 ± 1.7	63.7 ± 3.5	0.21	0.21

* P‐value from Kruskal–Wallis test

** P‐value from multivariable linear regression models containing gender and age as covariates.

## 4 Discussion

In the present study, we assessed the DNA methylation levels of the following cancer‐related genes: *iNOS*, *eNOS*, and *TP53*, in traditional smokers, HnB smokers and never smokers. In addition to its association with cancer development, LINE‐1 was also selected as a genome‐wide readout for methylation (Cordaux and Batzer 2009). To our knowledge, this is the first study to investigate DNA methylation in oral tissues taken from HnB smokers.

Although our results revealed no differences in the DNA methylation levels of *eNOS*, *TP53* and LINE‐1 between the three study groups, we found higher DNA methylation level of *iNOS* gene in HnB smokers in comparison to traditional smokers and never smokers.

This observed difference in *iNOS* methylation levels might be explained by the different levels of the generated toxicants between HnB smokers and traditional smokers. Although studies have shown that HnB cigarettes contain similar types of harmful constituents (e.g., volatile organic compounds, ‘VOCs’, and carbonyl compounds) as conventional cigarettes (Auer et al. 2017; Li et al. 2019; Uchiyama et al. 2018; Yu et al. 2022), they have demonstrated that the range of vegetable glycerin levels emitted from HnB products was higher than that emitted from conventional cigarettes, while the range of nicotine levels transferred by HnB products was lower than that by conventional cigarettes. In addition, although the amount generated from HnB products was small compared to those from conventional cigarettes, various kinds of VOCs, aldehydes, nanoparticles, and particulate matter were produced. Recently, Lim et al (Lim et al. 2023) characterized the VOC composition released by HnB cigarettes in vitro. The key VOC components released were identified as isoamyl acetate, limonene, and ethyl butyrate, with 2,3‐butanedinone exceeding the maximum daily intake limit according to the National Institute for Occupational Safety & Health (NIOSH) guidelines.

In our study, increased DNA methylation of *iNOS* was reported in HnB smokers, which could indicate a decreased expression of iNOS, in contrast to traditional smokers. Although increased iNOS activity has been positively correlated with different types of cancer (Thomsen et al. 1994; Pervin et al. 2010), its increased expression has been associated with favorable prognoses of some types of cancers (Puhakka et al. 2003; Anttila et al. 2007). These findings indicate the dual role of iNOS in cancers (Vannini et al. 2015), in which iNOS‐derived NO has tumorigenic or tumoricidal activities, which have been debated in cancer biology (Lechner et al. 2005; Singh and Gupta 2011). In fact, this might depend on the amount of NO, microenvironment (Vannini et al. 2015), and cell type. In this context, it was shown that epithelial cells in a growing tumor have tumorigenic properties via iNOS activity, while tumor‐associated macrophages have tumoricidal characteristics also via iNOS (Burke et al. 2013). Nonetheless, these dynamics tend to change over time (Burke et al. 2013), which could be attributed to the exposure time of cells to the generated NO. As such, these dynamics need to be elucidated in functional studies, and the implications of our findings regarding the increased DNA methylation of *iNOS* in the development of oral cancer in HnB smokers, in comparison to traditional smokers, need to be evaluated in future long‐term studies. It must be noted that smoking, whether traditionally or through HNB devices, is harmful, since the same toxicants are generated. However, a recent randomized controlled clinical trial showed that switching from traditional smoking to HnB smoking led to favorable changes in the same direction as smoking cessation, suggesting that switching to HnB smoking may reduce disease risk compared to continued traditional smoking (Lüdicke et al. 2019). This might be implicated in oral diseases as well, including oral cancer, but at this stage, this remains a mere speculation.

The results of our study need to be interpreted with caution as it has certain limitations; our cohort is of a small sample size and includes only Caucasians, as such, careful interpretation of the results must be taken into consideration, due to the potential ethnic influence over epigenetic modifications (Kwabi‐Addo et al. 2010; Straughen et al. 2015). Moreover, simultaneous analysis of DNA methylation and mRNA expression could have strengthened our findings.

## 5 Conclusions

Higher DNA methylation level of the *iNOS* gene was found in gingival tissues harvested from HnB smokers, while the DNA methylation levels of *eNOS*, *TP53,* and LINE‐1 were comparable across the three study groups. These findings suggest that HnB smoking could locally influence the epigenetic patterns in the oral mucosa, and differently from traditional smokers, which might be due to the different levels of generated toxicants between the two different types of smoking. However, further studies with a larger sample size and in the long term are needed to confirm and extrapolate the implications of our findings in terms of oral cancer development.

## Author Contributions

**Giulio Rasperini:** conceptualization, supervision. **Michele Codari:** writing – original draft preparation. **Alessandra Moiana:** investigation. **Letizia Tarantini:** methodology, writing – review and editing. **Valentina Bollati:** methodology. **Gionata Bellucci:** investigation. **Dario Consonni:** formal analysis, data curation, writing – review and editing. **Lena Larsson:** writing – original draft preparation. **Farah Asa'ad:** writing – original draft preparation, writing – review and editing.

## Conflicts of Interest

The authors declare no conflicts of interest.

## Data Availability

The data that support the findings of this study are available from the corresponding author upon reasonable request.
